# Evolutionary Image Registration: A Review

**DOI:** 10.3390/s23020967

**Published:** 2023-01-14

**Authors:** Cătălina-Lucia Cocianu, Cristian Răzvan Uscatu, Alexandru Daniel Stan

**Affiliations:** Department of Economic Informatics and Cybernetics, Bucharest University of Economic Studies, 010552 Bucharest, Romania

**Keywords:** evolutionary algorithms, image registration, fitness functions, image similarity indicators, accuracy index

## Abstract

Image registration is one of the most important image processing tools enabling recognition, classification, detection and other analysis tasks. Registration methods are used to solve a large variety of real-world problems, including remote sensing, computer vision, geophysics, medical image analysis, surveillance, and so on. In the last few years, nature-inspired algorithms and metaheuristics have been successfully used to address the image registration problem, becoming a solid alternative for direct optimization methods. The aim of this paper is to investigate and summarize a series of state-of-the-art works reporting evolutionary-based registration methods. The papers were selected using the PRISMA 2020 method. The reported algorithms are reviewed and compared in terms of evolutionary components, fitness function, image similarity measures and algorithm accuracy indexes used in the alignment process.

## 1. Introduction

The image registration process (sometimes called image alignment) encompasses a set of operations performed on pairs of images with the purpose of preparing them for further use in various applications, hence the general view of image registration as a preprocessing step. The involved images come from the same sensor or different sensors (in the same spectrum or different spectrums) and are captured at different moments in time, from different angles. Generally, they represent the same subject but are different in some way. The purpose of this kind of processing varies, but it usually involves applying a transformation on an image to make it align or match another. The goal can be enriching the informational content in an image by superimposing other images, stitching images together to create a larger image, the recreation of a 3D model, the detection and tracking of objects etc. The two images involved are usually called object (or target, reference image) and scene (detected, captured).

Two main approaches are used: feature registration and intensity registration. Feature registration means that some elements (salient objects) are detected in both images that go through the registration process, and a transformation is applied on the scene image such that selected elements match their correspondents in the object image. The features can be contours or points (landmarks), and they can be manually marked and paired or automatically detected. Manual marking is done by experts and obviously takes a lot more time and is prone to errors. The availability of experts can also be a problem. With the development of new algorithms and computer power, automated detection of features solves the problems raised by the need of human intervention. Feature registration requires less processing power since it uses few pixels in the image to compute the required transformation. In order to assess how well the images are aligned, various indicators were developed.

The increase in computer power allowed researchers to turn their attention towards intensity registration, which uses all the pixels in the images during the registration process. Since all pixels must be matched, the amount of computations is much higher, but more elements in the images are aligned. Specific indicators that take into account all the pixels were developed to assess the quality of the results.

Most digital images are 2D, so registration efforts naturally went into computing the parameters for a 2D transformation. Still, not everything is 2D. There are also 3D objects and efforts to compute 3D transformation to match such objects. The type of transformation can be rigid (geometric, affine), where all the pixels go through the same transformation, or free-form deformation.

The transformation parameters are vectors part of a large solution space, with infinite points (due to the real value nature of the parameters). Total exploration of such a space is impossible (it would require an infinite time), so researchers turned to stochastic algorithms that draw inspiration from biology. From the initial evolutionary algorithms (EAs) and genetic algorithms (GAs), a plethora of evolutionary algorithms of biological/nature inspiration were developed (and sometimes not quite biological but employing the same principles, i.e., the Fireworks Algorithm (FWA) [1]). Variation and selection operators were developed for each new algorithm and then researchers started mixing algorithms and operators in search for faster and/or more accurate algorithms. The evolutionary algorithms can use one individual (such as hill climbing or (1+1)ES, also called two-membered evolutionary strategy 2MES) or be population based (GA, ES, variants of swarm intelligence (SI)).

This is what drove us to find the current status and trends in solving image registration tasks using evolutionary approaches. We found that, as expected, evolutionary image registration is a very dynamic field, where advancements are supported by the rapid technological development. The main contributions of this paper are:

- identification of the most recent research papers in the field of evolutionary image registration, published in the last 5 years and indexed in major databases, available either openly from the publisher or the authors, or through the research network of which our university is a member;
- focus on successful use of evolutionary algorithms for image registration;
- identification of fields of application where research efforts are focused;
- main ideas of each reported research are summarized giving a clear view of the approach;
- identification and comparative analysis of the main (dis)similarity measures used to register images and performance indicators used to assess algorithms’ results and to compare them between algorithms;
- comparative analysis of main elements of evolutionary approaches: basic algorithm and algorithm class and fitness functions.

The rest of this paper is organized as follows: Section 2 presents the methodology used to select the articles for this review. Section 3 summarizes the selected articles. Section 4 discusses the elements identified in the review. Section 5 presents the conclusions.

## 2. Methodology

The review was conducted following the guidelines of the revised Preferred Reporting Items for Systematic Reviews and Meta-Analyses (PRISMA) [2]. The targets of this paper are articles published in the last five years (2018-present) that propose new or modified (improved) evolutionary algorithms or are used in an innovative way to solve registration problems. Additionally, the scope of the review is limited to articles indexed in international databases. The following databases were considered: Clarivate’s Web of Science database (WoS) [3], Elsevier’s Scopus database [4], IEEE Xplore [5] and Springer [6] databases. The review includes only articles that are available to us: either free access from the publisher or author or accessible through our university account on publishers’ sites.

Three searches were performed on the Web of Science site, using their own search engine [3]. The first search was performed using the keywords “evolutionary image registration”. Initial search results included 75 records, to which 80 authors have contributed. These records are distributed as follows: 59 articles, 14 conference proceeding papers, 2 reviews, 2 early access and 1 data paper. After screening titles and abstracts, 36 of the records were deemed to be relevant for the purpose of this review. The rest of the articles only coincidentally included the search terms, usually “evolutionary” in biology/medical articles and in 1 case the article was not relevant. Furthermore, 11 articles were eliminated because they were not accessible through our university account and 2 because they were reviews themselves. Six entries belonging to this review’s authors will be discussed together. Thus, 23 articles were kept for the purpose of this review. A second search used the keywords “bio-inspired image registration” and produced 14 records. Of these, 5 were duplicates of previous search results; 1 was a very similar article from the same author; 1 was a review; and the remaining 7 were not relevant. Thus, none of the 14 records added a new article to this review. A third search used the keywords “nature inspired image registration” and produced 16 records. Of these, 3 entries are considered relevant; 4 are duplicates of previously found articles; and 9 are not relevant, only coincidentally including one of the search terms.

A search was performed on the Scopus database using the keywords “evolutionary AND image AND registration”, yielding 70 records. Of these, 29 were duplicates of records already found in WoS. Twenty-five of the remaining articles were not relevant for the purpose of this review. Eleven more records pointed to articles that were not accessible (two of them because of the language in which they are written). One record was removed because it was a review. Four articles remained to be reviewed in this work.

The search on IEEE Xplore database using the criterion ‘(“All Metadata”:evolutionary) AND (“All Metadata”:image) AND (“All Metadata”:registration)’ yielded 20 records. Only 8 were new (not previously found in WoS or Scopus). Three of these articles were not relevant to the purpose of this review. Four more were not accessible to us. The 1 remaining article is included in this review.

The search on the Springer database was performed using the ‘“evolutionary” AND “image registration”’ search criterion and limited to full text accessible items. A total of 162 results were returned. After analyzing the titles, abstracts and the full text (where needed), only 4 of these records were retained as relevant articles not previously found in WoS or Scopus databases. The remaining results were either previously encountered (3 results) or not relevant (they were either reviews themselves, books/chapters of books/books of abstracts or only coincidentally contained the search terms).

## 3. Articles

In this section, the selected articles are briefly reviewed, mentioning the key elements and the idea underlying the proposed approaches. The articles are grouped considering the class of base algorithms used in the respective approaches.

### 3.1. Genetic Algorithm

Articles reviewed in this section use approaches based on the genetic algorithm (GA).

Pirpinia et al., 2019: In [7], an evolutionary algorithm is used in order to automate the process of deformable image registration (DIR). The target of the study is represented by medical images used in radiotherapy. The main goal is to replace the manual tuning of parameters used in DIR, which is performed in each individual case, with a big cost in time, with pre-established sets, determined using a multi-objective evolutionary algorithm. For each class of DIR problems, the evolutionary algorithm determines the best set of parameters that can be used to approximate the optimal solution. This set is used to solve all problems in that class. With a set of parameters available for each class, when an individual DIR problem has to be solved, a solution is computed for each set of predetermined parameters and an informed choice can be made from the set of results, which is much faster than trying to solve the DIR problem directly. An experienced practitioner can easily select the preferred result from the ones computed. The quality of the registration process is evaluated using two indicators: a dissimilarity indicator called negative normalized correlation coefficient (NNCC) [8] and an indicator describing the magnitude of deformation called bending energy penalty (BEP) [9]. The fitness function used in the evolutionary algorithm includes the two mentioned indicators and the sum of distances between landmarks in the target and corresponding aligned images. The evolutionary algorithm employed is an estimation-of-distribution algorithm (EDA) [10]. The quality of the algorithm results is evaluated by calculating the mean target registration error (mTRE) as the mean Euclidean distance between landmarks in corresponding pairs of target and aligned images. Parallel computing of several solutions, based on predetermined registration parameters, without manual tuning, followed by an informed choice between the presented results, was proved to be a less time-consuming solution, with good and usable results from a medical point of view. Although the algorithm itself is not new or improved, we deem this article worthy of this review due to the original approach in solving the DIR problem and the new way the evolutionary algorithm is used to automate solving a DIR problem.

Nakane et al., 2022: In [11], evolutionary algorithms are applied to estimate a free-form deformation model for pairs of images. The deformation model estimates the movement of a lattice of equally spaced control points overlayed on the image. In order to ease the complexity of calculations, the image is split in several regions (2 and 4) for which the deformation is estimated separately. Thus, the optimization problem is transformed in a multi-objective problem. The results obtained from the separate problems are combined afterwards to reach the final result. In addition, the algorithm uses a pyramid of images with increasing resolution: three levels of resolution, each level halving the resolution of the previous one. Computations start on the lowest resolution, using a correspondingly smaller lattice of control points. The results of each resolution level are used as entries for solving the problem on the next higher resolution, with the addition of more control points to the lattice. The evolutionary algorithm employed is a genetic algorithm (GA). The final population computed on a resolution level is used as the initial population on the next resolution level. Instead of computing the final result by aggregating the best individual in the four sub-problems (i.e., the one that leads to the best similarity for that region), the authors proposed a method that analyzes small groups of control points from those individuals and aggregates the groups that provide the best similarity for the local patch. The algorithm results are analyzed using the RMSE and mean Euclidean distance error (MEDE). The experimental results show that the dual objective problem gives better deformation estimation than the single objective problem, and the four objective problem is effective in estimating complex and subtle transformations.

### 3.2. Evolutionary Strategies and Swarm Intelligence

Articles reviewed in this section use approaches based on evolutionary strategies and various kinds of swarm intelligence algorithms.

Keikhosravi et al., 2020: In [12], the authors use evolutionary optimization to register pairs of medical images used in histopathology. The images come from two different imaging devices and contain complementary information that can help in biomedical research. One image is taken by the new second-harmonic generation (SHG) microscope. The second is the traditional bright-field (BF) image of hematoxylin and eosin (H&E) stained tissue. The images must be aligned to match the extracellular matrix (ECM) detected by SHG to the tissue depicted in BF. Since the images come from microscope samples, an affine transformation is considered, including rotation, translation and scaling. The H&E images contain a lot more information than the ECM and that can affect the registration process; therefore, a preprocessing stage eliminates the cell information from these images. Only the remaining ECM information is used for the registration with the SHG image. The similarity indicator used, which also serves as the fitness function in the evolutionary algorithm, is NMI. The evolutionary algorithm chosen is (1+1)ES. Microscope samples produce large images; therefore, a three-level pyramid image is used to achieve better computational speed, by reducing the resolution of the images on two more levels. The evolutionary algorithm is applied three times. First, it is applied on the lowest resolution image, starting with a random individual. Low resolution leads to high speed in calculation. The solution computed on this level is used as the initial individual when applying the optimization again on the mid-level resolution. In the same way, the solution produced by this stage is used as the initial solution for the last stage, where the optimization is applied on the original resolution images. Although the resolution is high, a low number of iterations is required since the initial individual is already in the vicinity of the optimal solution; thus, computation time is kept at a reasonable level. For verification, the results were compared with results of manual registration performed with manually selected landmarks. The pixel mean absolute error (MAE) indicator was used to compare the results of the two methodologies. Tests performed on a large number of image pairs led to a high success rate (over 92%) and in a good time. The unsuccessful attempts are not failures of the algorithm itself but due to factors affecting the images. These factors are discussed in the paper. The algorithm is also compared with state-of-the-art algorithms: SIFT-RANSAC [13], PSO-SIFT-RANSAC [14] and SimpleElastix [15]. For these particular kinds of images, none of the compared algorithms were able to achieve a high accuracy.

Vidal et al., 2022: In [16], the CMA-ES [17] algorithm is used to construct 3D CAD models from slices of X-ray scan of a material. The slices are automatically registered to create the final 3D model. The purpose of the study is to overcome the problems created by corrupted tomographic data (with artefacts) and downsides of existing methods, especially regarding the quality of the final result but also the time required to construct the model. The authors use an open-source implementation of CMA-ES. Four different image similarity measures are used to test which one performs better for the problem at hand: MAE, RMSE, ZNCC and DSSIM. The model created for this problem involves 23 parameters that have to be optimized. Some of the parameters are classical ones in registration problems. Due to the large number of parameters, it was not feasible to optimize them all at once, so they were split in several groups according to their meaning. The eight groups created were optimized sequentially, for seven of them evolutionary optimization being used. The results were compared with a user study, where volunteers manually registered the slices. The tests proved that the proposed method can automatically create the 3D model from the tomographic slice with high accuracy, allowing geometric analysis of the target object.

Bermejo et al., 2018: In [18], the registration of medical images is approached using the Coral Reef Optimization (CRO) [19,20] algorithm and its variant Coral Reef Optimization with Substrate Layers (CRO-SL) [20]. The authors chose a substrate layer (SL) combination consisting of five operators: crossover (BLX-α [21] and SBX [22]), evolutionary (HS [23] and DE [24]) and mutation (GM). In order to speed up the computations, a multiresolution strategy was used, with a small scale of images used to compute a rough estimate, and then a larger scale used to refine the initial results. The similarity metric used is NMI, which also plays the role of the fitness function for evaluation of individuals. Individuals encode the parameters of the 3D affine transformation necessary to align the pairs of images. The CRO and CRO-SL performance is compared against other evolutionary (SS) and classical algorithms (downhill simplex [25], Adaptive Stochastic Gradient Descent (ASDG) [26] and FLIRT [27]). The quality of the results produced by the compared algorithms were estimated using visual assessment and registration error as average distance between corners of volumes of interest (VOI). Execution time was compared for all involved algorithms. Additionally, statistical analysis was used, involving Friedman’s test [28], Bonferroni–Dunn’s test [29] and Holm’s test [30]. The experimental results ranked CRO-SL above the other algorithms and proved its accuracy and robustness.

Wu et al., 2019: In [31], the authors start from the Ant Colony Optimization (ACO) algorithm [32] extended to a continuous space (ACO_R_) [33] and add an adaptive multimodal component to create the Adaptive Multimodal Ant Colony Optimization (AM-ACO) algorithm. The main advantages sought from the continuous ACO are preservation of high diversity and good performance in multimodal optimization. The proposed solution partitions the population in clusters (niches) at each iteration, and each individual in each niche creates a new individual. The new solutions that are better than the best one in the current population go through a local optimization process where the energy function is minimized using the gradient descent method and the backtracking algorithm. Solutions optimized in this way are added to the population. The image similarity indicator is DTV, with MI also being used in some of the tests. The authors conducted tests on three pairs of images. In each test, the performance of the proposed algorithm was compared against other registration algorithms. RMSE was computed on 30 manually selected feature points to assess the performance. The test results indicate that the proposed algorithm is effective and robust.

Zhang et al., 2022: In [34], the authors set to improve the Squirrel Search Algorithm (SSA) [35]. The targeted downsides of SSA are related to premature convergence, population diversity and focus on local vs. global search. Elements of Quantum Particle Swarm Optimization (QPSO) [36,37] and an adaptive mutation mechanism are combined into the SSA. The proposed algorithm (AM-QSSA) is compared against 7 other bio-inspired optimization algorithms and state-of-the-art evolutionary algorithms, on 37 benchmark functions. Multiple tests show that AM-QSSA has better or comparable performance from the point of view of stability, efficiency, accuracy, success rate, convergence and complexity. As a real problem test, the algorithm was applied on the registration of medical images (MRI and CT). The similarity measure, which was also used as the fitness function, is NCC. The registration problem was solved using both SSA and the new AM-QSSA, with the new algorithm performing better than the standard one.

Bejinariu and Costin, 2018: In [38], the authors apply the Fireworks Algorithm (FWA) [1,39] to the problem of image registration. The similarity measure chosen for the study is NMI, which is also used as the fitness function. The accuracy of the results is assessed through NMI and NCC. The registration is performed under the assumption of affine perturbation. Tests were conducted using four images: two pictures, one graphic drawing and one pattern test image (combination of picture and graphic drawing). Images were degraded artificially so the registration results could be compared against the actual optimal transformation. The first test showed good results for three of the images but failed to register the graphic drawing. Two solutions for this failure are explored. One of them is to increase the values of parameters (number of generations and population size), which leads to unfeasible increase in the execution time. The second solution is to replace pixel-based registration with feature-based registration. The second test studied the influence of noise. The two pictures were used for this test: after applying the affine degradation, Gaussian or salt and pepper noise was added. The test showed good results, proving that the algorithm is insensitive to noise. The third test compared the results of FWA-based registration against three other evolutionary algorithms: PSO (as described in [40]), Cuckoo Search (CSA, as described in [41]) and GA (as described in [42]). Considering the accuracy and speed, the test indicated that each algorithm has its strengths and weaknesses: GA is faster in computing an acceptable solution; CSA is better at computing a precise solution but slower; while FWA and PSO are indicated for computing good solutions in reasonable time. One weakness is that all algorithms failed to register the graphic drawing test image. For this particular case, all algorithms were applied again using a different approach: feature registration instead of pixel registration. A small number of key-points were selected from the features extracted using SIFT, and the objective function was replaced with Euclidean distance between these pairs. With this approach, all algorithms were able to register the graphic drawing image.

Chen et al., 2018: In [43], the authors propose an improvement of the Bio-geography Based Optimization (BBO) [44] through the addition of new operators, adapted for the medical image registration problem. This improved algorithm is called Bio-geography Based Optimization with Elite Learning (BBO-EL). The first new operator, called hybrid full migration, replaces the original migration operator which is fast due to simplicity but limits the possibilities of exploration of the search space. The hybrid full migration operator is a mix of the BBO migration operator and DE operator. Additionally, the BBO immigration limit is removed to allow all good individuals to participate in the migration and enhance the exploration abilities of the algorithm. The second new operator reverses modifications done (undo operator) to an individual if those modifications reduce its quality, thus preventing the degradation of the lower limit of quality for the entire population. Quality degradation of an individual is a possible outcome of the fact that an individual receives information from many others, no matter their quality. The third new operator is called elite learning and is meant to improve the possibility of exploitation of a good area by involving more good individuals in the migration process. The elite learning operator is applied on the individuals that were not updated in the current iteration, allowing them to improve their quality. This leads to a higher upper limit of quality for the entire population and higher accuracy and convergence speed of the algorithm. The elite learning operator chosen for this algorithm is the PSO operator. The proposed algorithm is tested on medical images and its performance is assessed against state-of-the-art algorithms CRO, CRO-SL, BBO and improved Scatter Search (SS*) [45]. The tests showed that BBO-EL performs much better than the original BBO (which was worst of all used algorithms) and SS* and is even better than CRO-SL.

Saxenna and Pohit, 2022: In [46], the authors premiere the use of the Whale Optimization Algorithm (WOA) [47] for image registration. The problem approached in this article is the registration of near infrared (NIR) and visible spectrum images of the same subject to enrich the informational content available to analysts. MI was used as the similarity measure and fitness function. Additionally, MI was used to compare the results of WOA against other state-of-the-art algorithms: SURF [48], FAST [49], BRISK [50] and Harris feature detector [51]. Tests considered rigid transformations and were conducted on artificially degraded images with various parameters and included noisy infrared images. From the MI point of view, WOA obtained better results than all other compared algorithms.

Chen et al., 2022: In [52], the authors expand the registration performance of CRO by adding elements from other algorithms (FWA and DE), hence the name of the new algorithm FWBCR (Fireworks boosts Coral Reefs). The main idea of the newly proposed algorithm variant is to expand the exploration capabilities of CRO by adding the explosion operator from FWA. This improves the algorithm’s ability to escape the local optimum traps. In the reproduction stage of CRO (broadcast spawning), a small number of individuals with good fitness go through the explosion operator to produce many new individuals. The operator parameter (amplitude) determines how far new individuals are from the original one. In early stages, a larger amplitude is preferable to allow exploration of new areas of the search space. In later iterations, a lower amplitude is more suited to allow better exploitation of the neighborhood. Thus, the authors introduce a self-adaptive mechanism that correlates the amplitude with the quality of the population. Additionally, unlike in the regular FWA, the explosion is directed towards the most promising area in the search space. The orientation and adaptation of the amplitude are implemented by a mechanism inspired from DE: the differential migration vector (DMV). The DMV orients the explosion towards the optimal solution (most promising area) and adapts its size depending on the fitness of the best and least fitted individual in the population. Since a large number of new individuals created in the explosion increases the time consumed by the algorithm, only a few are kept as new individuals. The explosion results (sparks) are clustered, and only the cluster centers are kept, thus achieving a good balance between computational effort and diversity. The algorithm was tested against CRO-SL and BBO-EL. The similarity metric used to assess the results of the registration process is NMI, which also plays the role of fitness function. Tests showed that DMV is a good addition, improving the results obtained without it. From a visual point of view, FWBCR results are just as good as those of CRO-SL and BBO-EL. Analysis of the results showed that regarding the number of scenarios in which the best results were achieved, FWBCR performed better than CR-SL and almost as good as BBO-EL. The running time of FWBCR was instead much better than BBO-EL, a 30% gain being computed.

Shen et al., 2020: In [53], the authors propose an algorithm derived from the Flower Pollination Algorithm (FPA) [54] to register 3D cloud points. Traditional registration of 3D cloud points involves both translation and rotation on all three axes. On one hand, the search space for the rotation parameters is easy to set. On the other hand, the search space for the translation parameters is difficult to set and may require human intervention. The need for a broader translation search space (to make sure it includes the optimal solution) may make it difficult for the heuristic algorithms to find the optimal solution. The proposed method uses an evolutionary algorithm to find optimal values only for the rotation parameters, while the translation is computed directly for each member of the population, together with its fitness. To this end, data are normalized, as normal distribution is invariable to translation. Additionally, the search space for the rotation parameters is considered cyclic. The fitness function for the proposed method is based on the Pauta criterion, instead of the RMSE measure used in FPA. RMSE is used as the accuracy measurement to assess the performance of the proposed method against numerous other algorithms, both deterministic and evolutionary heuristics. Experiments were conducted on several sets of cloud points and proved that the proposed methods achieve very good results compared to the other algorithms in all cases, while some standard evolutionary algorithms are less likely to find a good solution, results being even inferior to deterministic local search algorithms in cases with complex initial position. Experimental results also show good registration accuracy in cases with noise and outliers. In some experiments, FPA was also used to refine the results of the proposed method. The proposed method was also tested for the registration of 3D models and quality control of surfaces in a real production environment.

Hu et al., 2020: In [55], the authors propose an improvement of the traditional Artificial Bee Colony (ABC) [56]. Since ABC is known to have a slow convergence rate, the authors endeavor to speed it up and balance the exploration and exploitation abilities of the algorithm. Two new elements are added to ABC: a leading group and a chaos operator. The leading group consists of individuals with good fitness values. The size of the group depends on the current iteration, being larger at the beginning of the search and decreasing to only one member at the last iteration. Bees in the population follow a randomly chosen member of the leading group, thus increasing the chances of converging to the optimal solution. The chaos operator is meant to extend the exploration ability of the algorithm, giving bees a chance to explore areas that are further away than the local spot. The proposed algorithm (LABC) was tested against numerous evolutionary algorithms on 12 benchmark functions. Using the convergence rate and mean and standard deviation of the results, the proposed algorithm was proved to be better. After validation, the algorithm was applied to image registration, where it was compared against four other algorithms. The MI was the chosen image similarity indicator and was used in the fitness function. The algorithms were compared on the basis of the mean and standard deviation of the achieved MI. The proposed algorithm achieved better results than the compared ones. Additionally, the proposed algorithm does not increase the computation complexity of the original ABC.

Liu et al., 2018: Manual work on the registration of images of various resolutions and captured by different types of sensors (i.e., satellite images) is time consuming and prone to errors. Additionally, traditional registration algorithms focus on affine transformations, excluding non-affine distortions often induced by various factors in satellite images. In [57], the authors design an automatic registration methodology, based on an evolutionary algorithm: PSO. The goal of this methodology is to match pairs of control points (CPs) from the target and sensed images. Traditionally, the control points are manually selected, which leads to errors. In the proposed methodology, the control points are selected by applying several automated algorithms. After the initial selection by the Harris detector [51], the list of points is refined and reduced using the local self-similarity (LSS) descriptor [58]. PSO is used to find the optimal combination of CPs (as pairs target-sensed image) while maximizing the MI. This approach can correct both rigid and non-rigid deformations. The proposed algorithm (LSS-MI) was compared to the traditional PSO algorithm, using MI as the similarity measure, and proved to produce better results. The effectiveness of the proposed algorithm was demonstrated by applying it to pairs of images with very different intensities and textures. Additionally, the proposed algorithm performance was compared against other standard algorithms. The comparison took into account the RMSE indicator and the number of correctly matched pairs of CPs. Both criteria showed that the proposed algorithm is superior in performance than the standard ones on the test images. The authors also discuss limitations of the algorithm and research still needed regarding the performance in other more challenging cases.

Wang and Yu, 2022: In [59], the authors explore the problem of registration between visual spectrum images and infrared images. The low resolution and inherent blur of infrared images create problems for registration algorithms. The authors propose improvements that can surpass these problems. First, the usefulness of NMI for image similarity is recognized, but in the problem at hand, applying NMI on the full images is counterproductive. Instead, authors apply NMI on saliency gradient images, naming the indicator SGNMI. The saliency gradient is computed for the entire visual spectrum image. For the infrared image, there is a preprocessing that includes segmentation into saliency and non-saliency areas; then, the saliency area is enhanced through a histogram equalization algorithm and the saliency gradient is computed. The NMI is applied on the two saliency gradient images. SGNMI reaches a maximum value when the two images are correctly registered, so the registration problem becomes a problem of maximizing the SGNMI. After defining the problem specific similarity measure, the authors propose an improvement of the Cat Swarm Algorithm (CSA) [60], called Chaos Cat Swarm Optimization (CCSO). This algorithm builds on CSA and attempts to overcome its premature convergence problem. To this end, a chaotic mapping is introduced. The authors’ choice for this element is a logistic chaotic mapping. It gives the algorithm a stronger exploration power and thus avoids the premature convergence. To test the proposed improvements, tests were carried out using CCSO with SGNMI as well as with two other similarity measures GWW-NMI [61] and SMI [62]. The registration results were compared using RMSE and MAE indicators. The tally of the results showed that SGNMI and GWW-NMI have similar mean MAE and RMSE over multiple runs, with SGNMI slightly slower due to the fact it performed additional computations regarding the saliency areas. SMI did not perform as well as the other two on any indicator.

Banharnsakun, 2018: In [63], the author adapts the ABC algorithm to the particular case of feature point matching used for image registration. Before the actual registration, the images are preprocessed to extract a set of significant feature points to be matched. To this end, the of-the-shelf Shi-Tomasi [64,65] corner detector is employed. NCC is used to determine if two points from the two respective sets are well matched. NCC is computed on the correlation windows centered on the two points. The fitness function used in the proposed algorithm (called ANC-NCC) is the summation of NCC values (SNCC) corresponding to each pair of points from the two sets. The proposed algorithm applies at each iteration the best registration parameters (scale and rotation) computed by ABC so far to the correlation window before computing the NCC for each pair of points. The proposed algorithm was experimentally compared with PSO and GA, both using NCC as the similarity indicator and both modified to perform double fitness function evaluations in order to match the ABC-NCC number of evaluations. The performance of the three algorithms was compared using two indicators: SNCC and Correct Point Pair Ratio (CPPR: number of correct pairs divided by total number of pairs). Both indicators pointed that ABC-NCC was a better algorithm than the compared ones, obtaining better bets and average values. Additionally, a lower standard deviation indicated ABC-NCC was a more robust algorithm. The convergence speed of the proposed algorithm was also better.

Cocianu et al., 2018–2022: In a series of articles [66,67,68,69,70,71], Cocianu et al. work on using evolutionary algorithms for the registration of images, considering rigid transformations (scaling on one direction/multiple directions). Their first paper, [66] (Stan and Cocianu, 2018), studies the use of the population-based Evolutionary Strategy (ES) [72] and the effect of various combinations of variation and selection operators. The study used mutual information ratio (MIR) as the similarity measure and fitness function. The quality of the results is evaluated using MIR, root mean square signal to noise ratio (RMS-SNR) and root mean square error (RMSE).

In the next proposal, [67] (Cocianu et al. 2019), ES is hybridized with the two-membered ES local search algorithm (2MES) [73]. In the first hybrid version, the ES is applied to compute a solution which is then enhanced through 2MES. In the second version, 2MES is incorporated into the ES to create a so-called memetic algorithm (MA). 2MES is used first to locally improve a small fraction of the initial population, ensuring a good start for the ES; then, at the end of each iteration but before selection of the next generation, if the best individual in the current population is not better (fitness-wise) than the current best individual, again a small fraction of the current population is locally improved using 2MES. Again, MIR is used as the similarity indicator and fitness function. The results are evaluated using MIR, SR, SNR and run time.

In the next evolution, [68] (Cocianu and Stan, 2019), the simplified version of FA, called Accelerated Particle Optimization (APSO), is added to fine-tune the registration parameters. FA and APSO variants are tested with new updating rules that improve their performance. For the registration methodology, ES is used to quickly compute a sub-optimal solution, and then APSO is applied to improve it. The resulting algorithm is denominated as ES-APSO. The similarity metric is MIR and the algorithms’ performances are compared using SR and SNR.

The algorithms above were applied for the automatic registration of black and white images of hand signatures. To reduce the computational effort, only object pixels in the image were used when decoding a candidate solution. The purpose was to automate the recognition of signatures, thus reducing the workload of persons that have to manually authenticate them. The proposed algorithms demonstrated a high success rate and validate the idea of integrating them in the information systems of organizations that need to process many signatures every day, such as banks.

The next studies explored the automatic registration of people’s faces. Images are again preprocessed into black and white contours and registration takes place on the object pixels to reduce the computational load.

In [69] (Cocianu et al. 2020), the study on using FA for image registration takes a new step, hybridizing FA with 2MES. A hybrid version uses FA to compute a good solution (as good as FA can find) and then 2MES fine-tunes that solution. A memetic version includes 2MES inside the FA iteration, where it locally improves a small number of randomly selected candidate solutions from the current population if the best member of the population is not better than the current best for the entire process. NMI is used as the similarity metric and fitness function. The performance of the algorithm is assessed based on the success rate and SNR. The proposed algorithms are tested against other significant algorithms, including PAT, with good results.

In [70] (Cocianu and Uscatu, 2021), the previously proposed algorithm is taken a step further. A first stage of the method computes the boundaries of the search space, thus adapting each execution of the algorithm to the input data. After that, the memetic algorithm proposed in the previous work searches for the best registration parameters. The fitness function is DICE. As a new improvement, at each iteration the population is divided into clusters. The number of clusters is dynamically adapted, based on the fitness of the current population. One member from each cluster is improved using 2MES. Furthermore, if a predetermined number of FA iterations go without improving the fitness of the best individual, a small number of individuals are replaced with new ones. The new individuals are randomly generated in the search space and improved through 2MES. The proposed algorithm is tested against well-known algorithms (namely Principal Axes Transformation (PAT) [74] and regular step gradient descent optimization (RS-GD) [75] using various metrics: SNR, peak SNR, DICE, Shanon NMI [76] and Tsallis NMI [77]. Tests showed a 100% success rate, and even more, correct registration was achieved even for heavily perturbed cases where PAT and EO (MATLAB one plus one evolutionary optimizer) failed.

In [71] (Cocianu and Uscatu, 2022), the registration method is further improved by the introduction of multi-scale images. After preprocessing the original images into black and white contours, they are reduced in scale with two separate factors. The smallest scale is used to produce an initial population of promising candidate solutions. Additionally, it is used in a procedure meant to avoid trapping the evolution into local optima. The larger of the two reduced scales is used to compute the registration parameters using the memetic FA-2MES algorithm. Tested against significant algorithms, the proposed methodology proved to be robust and accurate, correctly aligning images even where other algorithms failed.

### 3.3. Generic Evolutionary Algorithm

Articles reviewed in this section use variations of the generic evolutionary algorithm.

Spanakis et al., 2019: In [78], the authors introduce an enhanced version (EHAR) of the Harmony Search algorithm (HAR) [79], which is used for intensity-based image registration. Tests were performed on medical images and aerial terrain images. This suggests the algorithm could be applied for any kind of content. The enhancement is based on the addition of a machine learning algorithm to speed up the evolutionary search of the optimum solution. The computational complexity of the evolutionary algorithm, derived from the repeated evaluation of the fitness function, is overcome by approximating the fitness value instead of computing it for most iterations. The fitness function of an individual computes the Mutual Information [80] between the target image and the aligned image given by that individual. After testing several machine learning regression methods to approximate the value of the fitness function, the authors selected Support Vector Regression (SVR) [81] as the best among the tested ones. During the execution of EHAR, for every 100 iterations, a few actually compute the fitness function for new individuals while the rest use the approximated value given by SVR. The enhanced algorithm (EHAR) is proven through experimental results to be robust, successfully aligning the images while also doing so in a significantly shorter time than the original algorithm (HAR) and other test algorithms, both traditional (RSGD—regular step gradient descent) and evolutionary ((1+1)ES—one-plus-one evolutionary strategy).

Gomez et al., 2019: In [82], the application of evolutionary algorithms for skeletal identification is used. Evolutionary algorithms are used to automate the registration of ante-mortem and post-mortem images of the frontal sinuses with the purpose of identification of bodies. The authors use a real-coded evolutionary algorithm (RCEA): mean variance mapping optimizer (MVMO-SH). The similarity measure used to compare the target image and the aligned image is a variation of the DICE indicator called masked DICE [83]. This indicator is also used as the fitness function in the evolutionary algorithm. The evolutionary algorithm determines the best set of parameters for a 9-point degree of freedom transformation (3D translation and rotation, source-to-image distance and perspective distortion on X and Y axes). The performance of the algorithm is evaluated using two indicators: ground truth DICE (GT DICE) [84] and mean reprojection distance (mRPD) [85]. The second indicator was used to avoid any bias since masked DICE and GT DICE are correlated, being equal if there are no occlusions in the images. The algorithm proved to be robust and faster than the one used for comparison (differential evolution-DE [24,86]), being able to correctly match 80% of the samples in an automatic manner, thus greatly reducing the workload left for other matching methods.

Martinez-Rio et al., 2019: In [87], the authors propose a three-stage automatic registration of medical images, designed to replace the slow and tedious process of manual registration. The focus of the report is represented by retinal images obtained through two different imaging techniques: optical coherence tomography angiography (OCTA) and fluorescein angiography. Both methods produce useful results for diagnostics, but besides the common elements (main blood vessels), they also contain complementary elements that can be put together through registration for a better understanding of the pathology. In the first stage, both images go through a denoising process and segmentation of main blood vessels. In the second stage, an approximate registration is performed by template matching using the blood vessel information. This stage computes a rough approximation of the scaling factor. The third stage uses an evolutionary algorithm (DE, with the variant DE/best/1/bin [86]) to fine-tune the parameters of the affine transformation used to align the images. The initial population for the DE includes one individual that represents the rough transformation calculated in stage 2 and variations in its neighborhood. The fitness function used to evaluate individuals is zero normalized cross-correlation (ZNCC) [88]. The results show that application of DE led to an improvement of the rough results obtained after stage 2. The success rate was used to assess the performance of the method, and it was computed as 98.8%. Another verification compared the results obtained using only blood vessel information with the ones obtained using non-vessel information too. It proved that using only the significant information in the images (blood vessels) yielded better results. In order to mitigate the cost of running the population-based search, the authors also conducted a test in which image resolution was halved for the entire set before applying the registration process. In this case, the results were similar to the ones originally obtained, but the running time was almost halved, thus proving that a reduced resolution is a good choice when determining the registration parameters.

Sun et al., 2018: In [89], the authors propose an algorithm derived from DE, called SGD-DE, designed to achieve better registration of remote sensing images. The proposed algorithm divides the population in niches (clusters) and allows different evolution strategies to be applied on each niche. The idea of knowledge fusion [90] means that knowledge from different sources is used to create new knowledge. In the paper’s setting, it means that data from various niches is used to evolve the population. There are three types of knowledge used in various stages of the evolutionary algorithm. First, during mutation, information from the neighboring niche is used. Second, during selection, information about the best individual from every niche is used. Third, niche parameters are updated using information about the distances between the central values of each niche. Two functions are used to estimate the similarity of two images, one in each experiment: NMI and DTV [91]. The performance of the algorithms is evaluated using the peak ratio indicator. The proposed algorithm is compared against other evolutionary algorithms, based on DE and PSO, with the conclusion that it achieves better results regarding speed and registration accuracy, especially when using DTV as the similarity metric. The authors have also included a discussion about the influence of mutation strategy, selection strategy and niche size on the performance.

Bouter et al., 2021: In [92], the authors explore the possibilities of accelerating the speed of an evolutionary algorithm applied to a DIR problem. The speed advantage is gained by profiting on the parallelization potential of the Gene-pool Optimal Mixing Evolutionary Algorithm (GOMEA) [93] and its adaptation for Real Value genes RV-GOMEA [94]. Evaluation and evolution are performed in parallel groups of individuals that are independent: no individual in a group is correlated to an individual in another group. Additionally, the authors make use of a multi-scale strategy. Solving the DIR problem involves overlaying a triangular grid over the images. Four such grids are used, with increasing number of points. Computations start with the smallest scale. The population built after a predefined number of iterations is used to initialize the population for the next stage that uses the immediately larger scale and so on. The study shows that parallelization leads to a significant increase in the speed of the algorithm, opening the possibility of solving more complex registration problems, such as expanding the DIR problem from 2 to 3 dimensions.

Casella et al., 2019: In [95], the authors use a parallelized version of DE to explore the possibilities of speeding up the registration of images. The target of this study is the range image registration (RIR), where a 3D surface is reconstructed from several 2D images captured by single sensors from different angles. The study uses the Grid Closest Point (GCP) [96] transformation to make transformations easier. When the process involves aligning each pair of two consecutive images, it is called pair-wise registration, and each pair can be registered in parallel. The DE algorithm is used for the actual registration process of each pair, specifically a distributed implementation (dDE) developed by the authors, which is used with multiple populations: Asynchronous Adaptive Multi-Population Model for dDE (AsAMP-dDE) [97]. A topology is selected to represent the population. Each node in the topology contains a subpopulation and can send individuals to neighboring nodes. Each subpopulation evolves for a number of generations, after which individuals may be sent to the neighboring nodes. The evolution strategy used is DE/rand/1/bin [24]. Additionally, the control parameters of the subpopulations with better evolution are used for the subpopulations with weaker evolution to force them to explore new areas of the solution space. The similarity indicator used as the fitness function to evaluate the individuals is the mean square error (MSE). The test results were statistically analyzed using the Friedman, Quade and Aligned Friedman tests. The results proved that the algorithm is more robust and gives better quality solutions, while the time consumed is one order of magnitude lower than best algorithms used for comparison.

Wu et al., 2022: In [98], evolutionary multi-task optimization (EMTO) is used to register point clouds obtained from different angles in order to reconstruct 3D models. The derived algorithm, adapted for 3D point cloud registration, is called Multitasking Point Cloud Registration (MTPCR). Each pair of clouds is registered as a separate task and results are combined to obtain the 3D model. The separate tasks are not solved independently because of the potential to accumulate errors regarding the global consistency when combining the solutions. Instead, knowledge is shared between tasks so they solve each pair considering both local accuracy and the general consistency aspects. Solving the registration of each pair using both criteria (local accuracy and global consistency) can be a difficult job, and for this reason, the authors use an aiding task for each registration task. The aiding task solves the registration problem for a pair using only the local accuracy criterion. After each iteration, the aiding task and the main task share knowledge, represented by the best individuals in their respective populations. Although the amount of computation is increased by using the aiding task, the efficiency and accuracy are better, making it a worthy tradeoff. The proposed algorithm is compared against several well-known algorithms and the results are assessed from multiple points of view: registration accuracy (as rotation and translation error), robustness on noisy data, solution quality, efficiency and sensitivity to parameter setting, regarding the importance of global consistency in the registration of individual pairs. The analysis of the results shows the proposed algorithm performs better than the classic ones.

Carlos et al., 2022: In [99], the authors develop a methodology for registration of land images captured in various spectral domains by satellites. One direction of development is the use of a new similarity indicator based on histogram kernel predictability (HKP) [100]. The similarity metric, denominated SHKP, is meant to mitigate some of the disadvantages of NMI in two directions: better computation time and better estimation of similarity. The evolutionary algorithm employed is a variation of EA, called evolutionary centers algorithm (ECA). In this algorithm, new candidates are computed as variations of centers of mass of random groups of individuals from the current population. The idea behind this kind of evolution is to move individuals towards the points where the mass of the entire population is maximum. The use of random factors and random choice of the parent individual ensures the possibility of exploring all areas of the solution space. Another direction of development deals with parallelization of the algorithm, which is possible due to the fact it is based only on function evaluations. Furthermore, the algorithm is applied in two stages: the first stage computes a solution; then, the algorithm is applied again on a population of individuals derived from the solution computed by the first stage. The second stage has a low number of iterations to reduce the computation time. This is possible because the goal is to reach a finely-tuned solution starting from a population that is already close to the optimum. The authors performed numerous tests, comparing the proposed methodology with known algorithms. Parallelization proved to significantly reduce the computation time. One set of tests involved comparing the ECA and DE evolutionary algorithms, using both NMI and SHKP as similarity measures. The test suggests that SHKP is better than NMI, and ECA is better than DE. Another set of tests compared the proposed methodology against state-of-the-art methodologies in registration of multispectral images: HOPC, CFOG [101] and CoFSM [102]. The proposed methodology proved competitive, with similar or better results for most types of pairs of image specters.

Gomez et al., 2021: In [103], the authors build upon the evolutionary algorithm presented in [82]. The evolutionary algorithm is coupled with a neural network with the aim to automate as much as possible of the forensic specialists’ work in the identification of bodies using ante-mortem 2D radiographies and post-mortem 3D CTs (Computer Tomograph). In the first stage, a convolutional neural network (CNN) is used to segment the AM radiographic material in order to extract the region of interest corresponding to the frontal sinuses. For this stage, four X-NET [104] architectures were tested. In the second stage, the evolutionary algorithm MVMO-SH is used to register the 2D segmented images with projections of the 3D PM CT. The combination of the two stages results in an automated system that can be used for positive identification of skeletal remains. Experimental results showed that 50% of the cases can be automated, thus significantly reducing the need for manual comparisons and freeing the forensic expert for other tasks. Although this article does not introduce a new evolutionary algorithm or variant, it was deemed worth for this review due to the novel way of combining the previously developed evolutionary algorithm with convolutional networks to automate a real-world task.

Moravec, 2020: In [105], an evolutionary algorithm is applied to the problem of the biometric identification of people. The proposed algorithm, called eaICP, matches the contour of a scanned hand with the contour of a hand stored in database for identification. The algorithm is a combination of the classical iterative closest point (ICP) [106] algorithm and EPSDE [107] evolutionary algorithm. The two algorithms are intertwined, each generation of evolution being one iteration of ICP. The scanned images are preprocessed, from RGB to BW, and then fingers and knuckles are identified. A set of eight landmark points are determined for the analyzed hand. Stored data about people’s hands are also represented as sets of important points. The evolutionary algorithm tries to match the set of points determined from a scanned hand with a set of points in the database considering the following possible transformations: translation and rotation of the entire contour and rotation of individual fingers. The proposed algorithm is tested and compared against a large number of evolutionary algorithms belonging to evolutionary strategy, differential evolution, particle swarm optimization classes, genetic algorithms and polar bear optimization. The results are promising, indicating a very good accuracy for the proposed algorithm.

Abe et al., 2019: In [108], the authors use an evolutionary optimizer in an innovative way. Although details of the evolutionary algorithm are not provided, the application makes the article worth mentioning in this review. In order to reduce the radiation absorbed by patients during exploratory medical imaging procedures, a set of 2D fluoroscopy images are used to recreate the 3D motion of the target (the forearm). This replaces the need to expose the patient to multiple radiation scans. The 2D images are registered automatically considering a six degrees of freedom transformation (3D rotation and translation) by an evolutionary algorithm. Tests proved that the reconstructed motion has a high accuracy and can be used to diagnose articulation problems, such as dislocation and reduced motion.

Zhang et al., 2018: In [109], the authors adapt the DE algorithm in order to achieve the registration of 3D cloud points that partially overlap. Starting from the fact that traditional algorithms may fail in case of partial overlapping, especially if the overlapping rate is low, the authors focused on an evolutionary algorithm that can better explore the search space and escape local optimum areas. The DE is the algorithm selected, with modifications made to the mutation operator. The mutation includes a self-adaptive mechanism that differentiates the mutation effect on individuals based on their fitness and the relation with the best fitness in the population. The fitness function is based on the Euclidean distance between pairs of points in the two clouds. The proposed algorithm is compared against a particle filter (PF) [110], genetic algorithm (GA) [111] and original DE. Tests results show that the proposed algorithm has slightly better results than GA, both being better than the other two algorithms. The success rate of the proposed algorithm was better than all the other considered algorithms on all tests.

Fischer et al., 2018: In [112], the authors explore the use of an evolutionary algorithm to automatically register images captured by different satellite sensors (optical and synthetic aperture radar-SAR). Correct registration of these images improves the geometric accuracy of the optical images and reduces alignment errors. The authors use the canonical EA, modified by adding a local search at the end of each iteration. The steepest ascent hill climbing algorithm is used to locally improve the best individual of the population if it has a fitness above a threshold. Tests performed using satellite images showed that the algorithm has a high success rate in aligning the images and can be used to automate the process.

## 4. Discussion

Throughout the reviewed articles, there is a large variety of algorithms, approaches and applications. In the following part, we will summarize the basic evolutionary algorithms used directly or improved, the similarity measures, fitness functions, performance indicators and fields of applications. In this section, the articles are identified by the subsection number where they were reviewed and their authors.

### 4.1. Field of Application

Image registration is a preprocessing procedure used in many fields, including medical imaging to support diagnostics and treatment, weather forecasting, land and crop survey, aerial terrestrial imaging, 3D modeling, object detection and tracking, computer vision, artificial intelligence, etc. In the reviewed articles, the medical field raises the highest interest, with many articles presenting applications on the registration of radiographies, CTs and MRIs.

Table 1 presents the fields where the reviewed article applied the registration algorithms. Note that some of the articles do not mention a specific field, i.e., they propose general purpose algorithms; therefore, they will appear as unspecified. Additionally, some articles use images from multiples fields to test the proposed algorithms. The medical field includes radiographs, CTs, MRIs and all similar imaging techniques. The geographical field includes aerial and satellite imagery captured with various types of sensors (visible spectrum optical, near infrared (NIR), radar, etc.). The 3D models field refers to 3D clouds of points and 2D CT slices used to build 3D models of objects. The field noted as identification refers to the identification of various body parts (hand, face) with application in biometric identification. The identification of inanimate objects (i.e., signatures) is considered separately.

**Figure 1:**
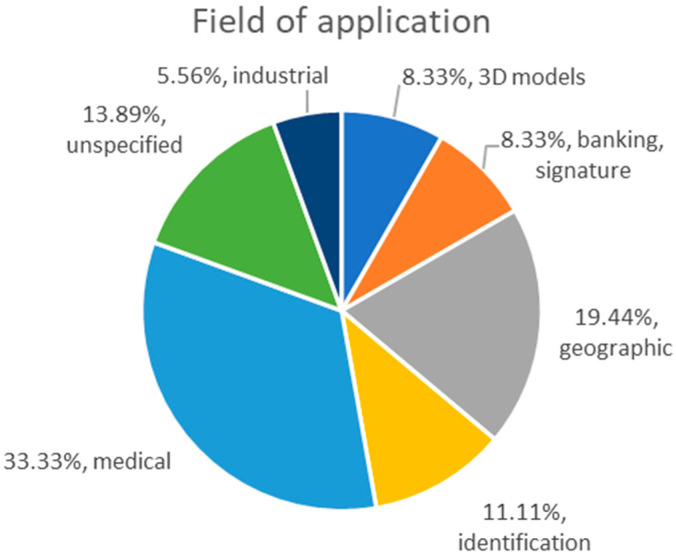
Repartition of fields of application in reviewed articles.

The repartition of fields of application (Figure 1) shows that the medical field is the main target of efforts to develop faster and more accurate evolutionary registration algorithms to ease and improve the diagnostic process or forensic identification. It is followed by the geographic field that matches aerial/satellite imagery to enhance the informational content or create larger images from smaller sections individually captured by sensors. Together, these two fields make up the bulk of the research effort.

### 4.2. Basic Evolutionary Algorithm

Most reviewed articles develop basic evolutionary algorithms by hybridizing or adding new interpretations and elements. Whether basic algorithms are applied, or new ones are developed, one thing is common to all: the parameters to be optimized are real values, so only evolutionary algorithms originally designed or later adapted to work with this kind of value are used.

Table 2 presents the basic algorithms encountered in the reviewed articles. Note that some of the articles combine elements from more than one algorithm. Additionally, (1+1)ES and 2MES are two alternative names designating the same algorithm.

It is noticeable that population-based optimization is the solution of choice for researchers. Evolutionary algorithms that use a single individual are mainly secondary elements used to locally improve some members of the population in order to increase the accuracy. Only incidentally does such an algorithm play the main role in registration.

**Figure 2:**
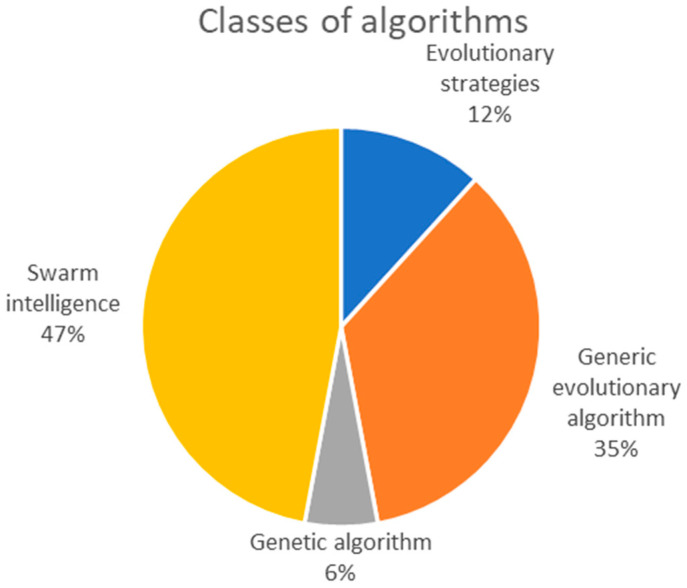
Repartition of main classes of algorithms in reviewed articles.

Figure 2 shows the repartition of algorithms into main classes. Various variants of swarm intelligence and evolutionary algorithms are the preferred approach of the researchers, while genetic algorithms and evolutionary strategies form a small part.

Most algorithms are hybrid methods consisting of a main population-based search algorithm combined with a local search procedure. The purpose of the hybridization is to ensure a good balance between the exploration of the search space and the exploitation of good candidates, thus increasing the chances of finding the global optimum solution. Considering the focus of the reviewed articles, SI-based methods appear to have better success than other types of evolutionary algorithms.

### 4.3. Similarity Measures

Many similarity measures were developed to assess how close one image is to another. Some use all the pixels in the image; some use only some selected pixels (features, landmarks). The choice obviously depends on the type of registration: intensity registration (which uses all the pixels in the image) or feature registration (which uses only some pixels). There are algorithms that use only some pixels to compute the registration parameters and all the pixels to estimate the (dis)similarity of the images. The most used classes of indicators are listed in Table 3.

**Figure 3:**
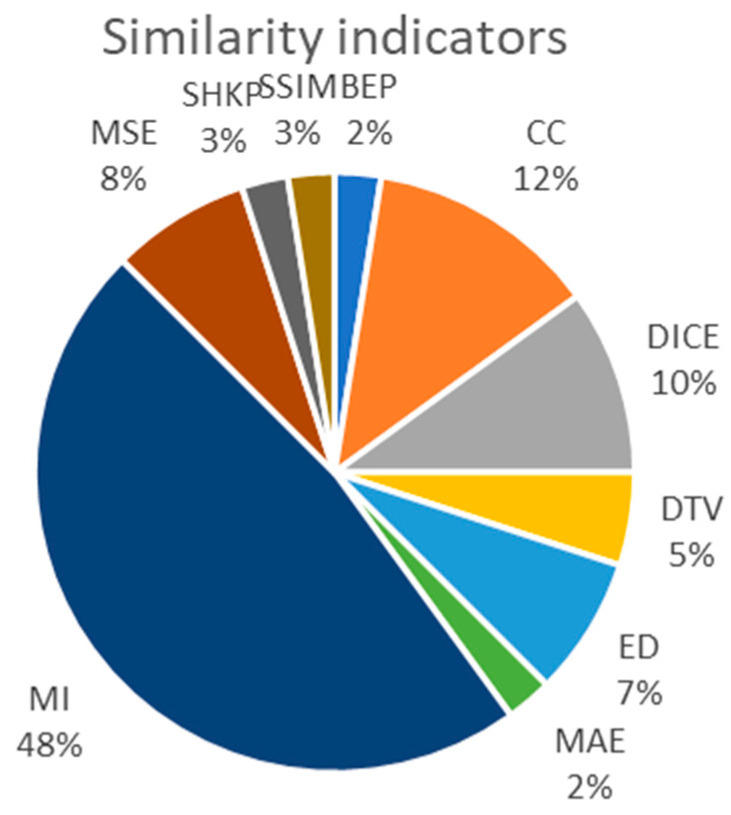
Repartition of similarity indicators used in reviewed articles.

Figure 3 presents the repartition of choices regarding the similarity indicators used in the reviewed articles. For this repartition, the indicators were grouped, considering that NMI and MIR are variants of MI, masked DICE is a version of DICE, MEDE is a version of ED and ZNCC, NCC and 1-ZNCC are versions of CC. The chart demonstrates a strong preference for MI-type indicators, while DICE, CC and pixel error (MAE and RMSE) are also used. The Euclidean distance is used in studies that perform the registration on selected features of the images.

In addition to the standard SSIM and SNR class of measures used in image processing, various similarity/dissimilarity measures have been used either to define the fitness function or to assess the performance of the obtained algorithm, each with its own strengths and weaknesses.

In feature-based methods, the similarity functions usually measure the distance between corresponding features [113]. For instance, if the features are points, the alignment can be evaluated using the (root) mean square error (RMSE, MSE) between the position of a point in the model and that of the corresponding (or closest) point in the transformed scene. Unlike MAE, both MSE and RMSE are used to focus on large errors; therefore, they are able to detect outliers.

Intensity-based approaches are usually developed using measures that can capture the resemblance of the intensity values in images depending on what kind of relationship is established between their intensity distributions (linear or non-linear) [113]. ED is the simplest solution to the problem of measuring the (dis)similarity between two vectors. Usually, the distance measure has qualitatively the same behavior as the correlation between two vectors. Additionally, the correlation performs well if the images differ only by simple noise. The correlation coefficient (CC) performs well for an affine relationship between pixel values. NCC is a cross-correlation function normalized by the standard deviations in [−1,1]. Our study reveals that the class of CC-based similarities is frequently used in medical image registration as well as for 3D models. The CC-based measures are relatively efficient since their computational complexity is linear in terms of the image size, but they are only suitable for determining the similarity between images with linearly related intensities. When images are such that their corresponding intensities are nonlinearly related, perfectly matching images may not produce high enough CC values, causing mismatches. Moreover, NCC is known to be sensitive to outlier information [114].

In case of multi-modal registration, the relationship between the intensity values in the images is usually non-linear. Metrics based on information theory, such as mutual information (MI), are better suited for this scenario. Shannon mutual information is widely used in image registration, but it is sensitive to noise. To reduce the influence of outliers, one can use similarity measures based on Tsallis entropy instead. The metric MI can be normalized by the joint entropy [115,116]. The obtained measure, NMI, is bounded between 0 and 1. Note that since the search landscape of NMI proved more appropriate for evolutionary computation algorithms than simple MI, symmetric uncertainty is very suited to measure the similarity between two images. The complexity of MI-based measures can be defined in terms of the number of bins (grey levels) within each image and in terms of the image size. However, since the number of bins of the histogram is much smaller than the number of pixels within the image, the computational complexity of MI similarities is linear in image size.

Finally, in case of binary images, one can represent data using vectors of object pixels instead of intensity matrices. For this type of dataset, simpler and more efficient similarity measures, for instance the DICE coefficient, can be used.

### 4.4. Fitness Functions

All evolutionary algorithms make use of a fitness function that evaluates the quality of each and every candidate solution. The fitness is used, depending on the type of algorithm, to select parents for the creation of new individuals and survivors for the next generation. In image registration problems, the fitness is usually based on the similarity metric used by the algorithm, often being one such indicator. Some studies ran experiments using several fitness functions to compare the results and decide which one is more suited for each task. Table 4 presents the fitness function classes used in the articles selected for this review. Since the fitness is based on the similarity indicators, the same conclusion arises: mutual information is the preferred measure, followed by DICE and Euclidean distance and pixel error.

There is a large variety of problems, using many different algorithms and (dis)similarity measures, making it impossible to name only one fitness function as a general recommendation. The fitness functions are based on the (dis)similarity indicators and aim to maximize either one such indicator or minimize in case of dissimilarity. For binary representations, simpler similarity measures are indicated, such as DICE. For more complex images, informational-based indicators, for instance NMI, are in most cases better suited. In cases where pairs of feature points are used for registration, Euclidean distance and CC are commonly used choices.

### 4.5. Performance Indicators

The performance of algorithms must be compared in order decide if a proposal is an improvement or to decide which algorithm is better suited for a specific task. Various performance indicators can be used for this purpose. Usually, the criteria used are related to robustness/success rate (correct registration was achieved or not), time consumed (either clock time or number of iterations) and accuracy of registration (various error measures). A large variety of performance indicators are used, demonstrating the preoccupation of researchers to thoroughly analyze the algorithms and their applicability to real registration problems.

Table 5 presents a summary of performance indicator classes used in the reviewed articles.

Throughout the reviewed articles, there is a large variety of algorithms, approaches and applications. All authors report improved performance compared to basic and classical/state-of-the-art algorithms. Performances are measured in various ways, using various indicators, depending on the field of application and the purpose of the research. Many researchers report the results in terms of success rate. In some cases, the success rate is defined as the recognition capability, the algorithms proposed in [57,66,67,68,69,70,71,78,105,112] achieving a maximum rate of 100%. Some articles analyze the robustness of the algorithms against additive/blurry/motion noise, the success rate reaching 96% in [59] (48 of 50 pairs correctly matched) and 98.8% in [87]. Some research automates the identification of medical images (forensic identification, histopathology tissue samples, 3D model reconstruction), with high success rates that translate into a large reduction of manual work by specialists [12,16,82,103]. For algorithms that attempt to match pairs of feature points, a measure of performance is given by the mean distance between the paired points, in the target image and transformed sensed image. For this type of performance measure, encouraging results have been reported in [18,87,92]. Additionally, good results are reported in [43,52] regarding registration of different types of medical images to enhance the informational content. A total of 54 multi-modal scenarios were analyzed using state-of-the-art algorithms and the proposed ones. The BBO-EL algorithm performed best in most scenarios (30), while FWBCR performed almost as good but was 30% faster. Most researchers reported better execution time for the proposed algorithm, as compared to original and classical/state-of-the-art algorithms. Population-based algorithms are inherently time consuming, but they produce more accurate results. Hybridization of algorithms aims in improving the execution time and was proven to manage that. Parallelization of algorithms also achieved better execution times, in some cases the improvement being dramatic.

As a conclusion regarding the performance indicators, besides standard image processing measures (such as SNR, RMSE, MAE and SSIM), the specifics of stochastic methods for image registration impose the use of recognition rate, success rate and average Euclidean distance between pairs of feature points. The recognition/success rates are closely related to the values of similarity measures used to define the fitness functions, as success means achieving a similarity measure above a predefined threshold. This is why normalized similarity measures are recommended: the optimum value is known in advance, and a threshold can be defined.

## 5. Conclusions

The goal of this review is to find out what are the current trends in regards to the use of evolutionary algorithms in solving image registration problems. We set out to find what are the latest developments and hot areas regarding the fields of application, type of algorithms, image (dis)similarity measures and fitness functions. Additionally, we studied what performance indicators are considered when comparing various algorithms. We wanted to find out what are the recent advances and promising research directions for the future. In our opinion, this review is a very good tool for other researchers that enter this field and pursue the development of computer vision components and systems.

We found that, as expected, evolutionary image registration is a very dynamic field, where advancements are supported by the rapid technological development. Population-based evolutionary research is resource intensive, requiring lots of computations, especially for the fitness function. A huge number of fitness function evaluations is the main consumer of time and obstacle in achieving a fast and accurate registration. Technological development mitigates this problem with ever faster processors with multiple cores and the possibility of parallelization. This also permits more tests to be carried out, both to compare algorithms on a specific problem and/or data set and also to compare the behavior of a specific algorithm and combination of elements on multiple data sets and problem variations. The decreasing cost of fast computers also allows more and more researchers to enter this field without limitations regarding access to computer power.

The reviewed articles demonstrated that new algorithms and algorithm variants are continuously proposed and explored. Algorithm components, such as variation operators, are also developed, either as new operators or adapted to the specific problem. Operators are tested in new combinations, sometimes surprising ones. The main target of the research regarding variation operators is to find a better balance between exploration of the search space and exploitation of good individuals. Exploration is intended to look for good solutions in all areas of the search space, which is especially important for multimodal functions. Exploitation is intended to refine good individuals and find the most accurate solution possible.

The availability of computing power also supports the move towards intensity-based registration. While feature-based registration is concerned with only some elements of the images processed, intensity-based registration works on all pixels in the image. Obviously, a huge amount of calculations is required to determine the similarity of two images by involving all the pixels, and computer power was a powerful limitation. Now, this barrier is falling and the path for exploration is open. New indicators are developed and classical ones are adapted, either to make them more useful or easier to use. From the multitude of similarity indicators developed, the reviewed articles show a preference for the ones based on mutual information.

Tests carried out on various algorithms showed that while improvements are achieved in robustness, accuracy and time consumed, there are variations regarding the performance between datasets. A universally appliable algorithm combination is yet to be found, so the path is open for further exploration.

We conclude that this review is very useful for all researchers that delve into the field of evolutionary image registration because it surveys in detail the main approaches of all recently published and indexed research papers. It points specialists to hot areas and also very promising research directions. Additionally, it presents all the elements that must be used to develop fast and accurate real-world applications in various fields that take advantage of image registration capabilities.

## Figures and Tables

**Table 1:** Fields of application in the reviewed articles.

Articles	Field of Application
[16,98,109]	3D models
[66,67,68]	banking, signature
[31,46,57,78,89,99,112]	geographic
[69,70,71,105]	identification
[53,59]	industrial
[7,12,18,34,43,52,55,78,82,87,103,108]	medical
[11,38,63,92,95]	unspecified

**Table 2:** Basic algorithms used in reviewed articles.

Articles	Basic Algorithm(s)	Type *	Class **
[7]	GA	Population	GA
[11]	GA	Population	GA
[12]	(1+1)ES	Single individual	ES
[16]	CMA-ES	Population	ES
[18]	CRO	Population	SI
[31]	ACO	Population	SI
[34]	SSA, QPSO	Population	SI
[38]	FWA	Population	SI
[43]	BBO, DE	Population	SI
[46]	WOA	Population	SI
[52]	FWA, CRO	Population	SI
[53]	FPA	Population	SI
[55]	ABC	Population	SI
[57]	PSO	Population	SI
[59]	CSA	Population	SI
[63]	ABC	Population	SI
[66]	ES	Population	ES
[67]	ES, 2MES	Population	ES
[68]	APSO, ES	Population	SI
[69]	FA, 2MES	Population	SI
[70]	FA, 2MES	Population	SI
[71]	FA, 2MES	Population	SI
[78]	HAR, SVR	Population	G-EA
[82]	RCEA	Population	G-EA
[87]	DE	Population	G-EA
[89]	DE	Population	G-EA
[92]	GOMEA	Population	G-EA
[95]	DE	Population	G-EA
[98]	EMTO	Population	G-EA
[99]	EA	Population	G-EA
[103]	RCEA	Population	G-EA
[105]	ICP, DE	Population	G-EA
[108]	unspecified	Population	-
[109]	DE	Population	G-EA
[112]	EA, Hill climbing	Population	G-EA

* Type indicates whether the main evolutionary algorithm maintains a population of individuals or works with only one individual. Note that some proposals include a local optimization algorithm that works with only one individual (Hill climbing, 2MES). ** Class indicates the category for the main algorithm: G-EA—Generic Evolutionary Algorithm (unspecified details), GA—Genetic Algorithm, ES—Evolutionary Strategies, SI—Swarm Intelligence.

**Table 3:** Similarity indicators used in the reviewed articles.

Articles	Image Similarity
[12,18,31,38,43,46,52,55,57,59,66,67,68,69,78,89,112]	MI-based
[11,16,95]	MSE-based
[99]	SHKP
[16]	SSIM
[7]	BEP
[7,16,34,63,87]	CC-based
[70,71,82,103]	DICE-based
[31,89]	DTV
[11,98,109]	ED-based
[16]	MAE
[53,92,105,108]	unspecified

**Table 4:** Fitness functions used in the reviewed articles.

Articles	Fitness
[12,18,38,43,46,52,55,57,66,67,68,69,78,89,112]	MI-based
[16,34,63,87]	CC-based
[70,71,82,103]	DICE-based
[16,53,95]	MSE-based
[7,38,109]	ED-based
[11,31,53,59,92,98,99,105,108]	others

**Table 5:** Performance indicators used in the reviewed articles.

Articles	Algorithm Accuracy
[11,12,31,53,57,59,66,67,68,69,70,71,78,89,99]	Standard image processing error/similarity measures (SNR/SNPR, RMSE, MAE, SSIM)
[57,63,67,68,69,70,71,87,109]	Success/recognition rate
[38,43,46,52,55,66,67,70,71,99]	MI-based
[34,38,63],	CC-based
[7,11,82,92,98,103,105]	ED-based
[70,82,103]	DICE-based
[18]	average distance of corner VOI, Bohmann–Dunn test, Holm test, visual
[95]	Friedman, Quade, Aligned Friedman
[16,108,112]	unspecified

## Data Availability

Not applicable.
